# An innovative treatment for hypoxic–ischemic encephalopathy: Silk fibroin nanomaterials improve neural stem cell axon formation and facilitate cognitive improvement

**DOI:** 10.4103/NRR.NRR-D-24-01178

**Published:** 2025-06-19

**Authors:** Chao Han, Shuna Chen, Zihan Shi, Xin Guan, Wei Zou, Jing Liu

**Affiliations:** 1 Stem Cell Clinical Research Center, National Joint Engineering Laboratory, Regenerative Medicine Center, The First Affiliated Hospital of Dalian Medical University, Dalian, Liaoning Province, China; 2 Dalian Innovation Institute of Stem Cell and Precision Medicine, Dalian, Liaoning Province, China; 3 College of Integrated Chinese and Western Medicine, Dalian Medical University, Dalian, Liaoning Province, China; 4 Department of Neurology, The First Affiliated Hospital of Dalian Medical University, Dalian, Liaoning Province, China

**Keywords:** astrocyte deactivation, axonal development, cognitive recovery, hypoxic–ischemic brain injury, motor function, nerve regeneration, neural stem cells, neurotrophic factor, silk fibroin hydrogel, synapse formation

## Abstract

Stem cell therapy shows promise for treating brain injuries; neural stem cells in particular are capable of repairing damage by forming new nerve cells and supporting recovery. However, optimizing the implantation and functionality of these cells in damaged brain regions remains challenging. Silk fibroin, a natural protein derived from silkworm silk, is a biocompatible material with exceptional properties that are useful for tissue engineering. Its biodegradability, mechanical robustness, and ability to promote cell growth make it particularly valuable for biomedical applications. Silk fibroin nanomaterials, which comprise silk fibroin processed into nanostructures, offer enhanced surface area, improved loading capacity for bioactive molecules, and superior nanoscale interactions with cells compared with bulk silk fibroin materials. In this study, we first extracted human-derived neural stem cells from a 14-week-old human fetus. Then, neural stem cells were loaded with 1% silk fibroin nanomaterials, which was identified as the optimal concentration to support human-derived neural stem cell growth and release of neurotrophic factors. Finally, 1% silk fibroin nanomaterials were implanted into a rat model of hypoxic-ischemic brain injury. The results showed that, compared with the treatment with human-derived neural stem cells alone, silk fibroin hydrogel carrying human-derived neural stem cells was significantly more effective at alleviating brain tissue damage, increasing neurotrophic factor secretion in the brain microenvironment, and promoting motor and cognitive function recovery. These findings suggest that silk fibroin nanomaterials loaded with human-derived neural stem cells could be used to treat hypoxic-ischemic encephalopathy. However, the mechanisms and related signaling pathways by which hydrogels combined with cells exert their reparative effects still require further in-depth investigation.

## Graphical abstract

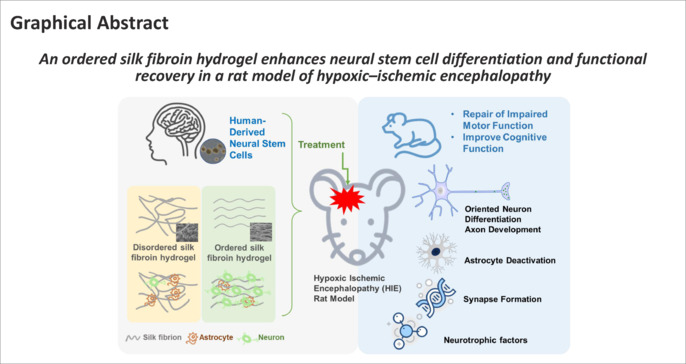

## Introduction

Hypoxic-ischemic encephalopathy (HIE) is a neonatal brain injury resulting from interrupted blood flow and oxygen supply before, during, or shortly after birth (Yang et al., 2025). It leads to progressive brain tissue loss and impaired neurogenesis, particularly in the hippocampus and cortex, resulting in long-term motor and cognitive dysfunction (Ehlting et al., 2022; Liu et al., 2023). The loss of neurons is typically permanent and irreparable because of the limited regenerative potential of neurons, the disruption of neural circuits, and the complexity of the brain’s structural organization (Liaudanskaya et al., 2019; Ebrahimi et al., 2021). Consequently, the development of therapeutic strategies for functionally restoring damaged nerves has become a key focus of tissue engineering.

Neural stem cells (NSCs) are emerging as a promising treatment for HIE because they offer both tissue replacement and neuroprotection (Huang and Zhang, 2019; Tang et al., 2024). Studies show that grafted NSCs can differentiate into neurons, oligodendrocytes, and astrocytes, with differentiated neurons demonstrating functional synaptic integration (Daadi et al., 2009; Tsupykov et al., 2014). NSC transplantation also enhances endogenous repair by modulating microglial activity, promoting axonal sprouting, and reducing neuronal apoptosis (Daadi et al., 2010; Guo et al., 2020). Additionally, NSCs secrete neurotrophic factors such as brain-derived neurotrophic factor (BDNF) and nerve growth factor (NGF), which improve neuroplasticity (Lee et al., 2017; Jiang et al., 2019). Clinical trials have shown that NSC implantation improves long-term neurological function and promotes motor and cognitive recovery (Kalladka et al., 2016; Muir et al., 2020; Lv et al., 2023). However, the adverse microenvironment created by hypoxic–ischemic brain injury limits the survival, differentiation, and integration of transplanted NSCs, often reducing their effectiveness (Huang and Zhang, 2019).

Rapid advancements in tissue engineering have made it feasible to assemble cells, tissue transplants, and biological materials into composite scaffolds (Xu et al., 2021). The physical and chemical properties of these scaffolds help regulate cell behavior and organization, creating a favorable environment for repairing damaged brain regions (Singh et al., 2019). Advances in tissue engineering techniques, particularly hydrogel-based three-dimensional scaffold printing, have resulted in the ability to manufacture materials that closely match the mechanical properties of brain tissue and can be combined with cell therapy for nerve injury repair (Cadena et al., 2021; Li et al., 2021). Silk fibroin (SF) is an ideal material for hydrogel scaffolds because of its biocompatibility, biodegradability, and plasticity (Yonesi et al., 2021; Semmler et al., 2023), and has been used in neural disease treatment (Koffler et al., 2019; De Vitis et al., 2024). The topology of scaffolds directly influences cell adhesion, growth, migration, and differentiation (Mattiassi et al., 2023). Recent advances in SF nanomaterial fabrication enable scaffold structural modifications that enhance nerve cell growth and tissue repair (Tan et al., 2015; Wu et al., 2018; Jiang et al., 2020; Xu et al., 2024). Among existing manufacturing methods, electrospinning provides a cost-effective approach to produce highly aligned fibers and mechanically stable membranes (Zhu et al., 2024). The resulting electrospun silk further integrates inherent silk advantages with improved surface area, porosity, and air permeability (Dai et al., 2024).

Here, we synthesized SF hydrogel scaffolds with ordered topological structures using electrostatic interactions to serve as a growth matrix for hNSCs. The aim of this comprehensive preclinical study was to determine whether electrospun SF hydrogel scaffolds enhanced the ability of human NSCs (hNSCs) to treat HIE. Furthermore, we compared the efficacy of hNSCs transplanted with or without SF hydrogel scaffolds in alleviating HIE damage in a rat model.

## Methods

### Silk fibroin hydrogel preparation and nanofiber fabrication

**Figure 1:**
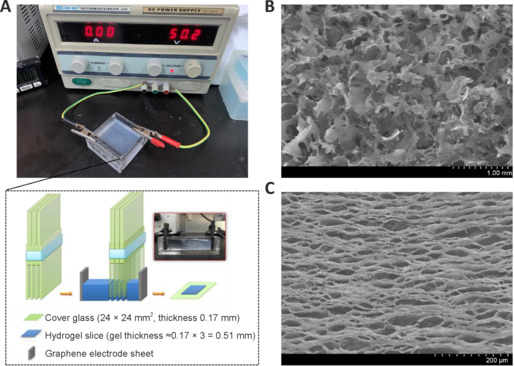
SF hydrogel preparation and characterization. (A) Hydrogel formation under an electric field. (B) Scanning electron microscopy images of SF hydrogels not subjected to an electric field, showing disordered nanofiber alignment (30× original magnification, scale bar: 1 mm). (C) Formation of oriented layers induced by electric field treatment. SEM images showing aligned nanofibers within the hydrogel structure (200× original magnification, scale bar: 200 μm). SF: Silk fibroin.

Aqueous-derived SF solutions were prepared as previously described (Wei et al., 2018). Briefly, a 6% (w/v) SF solution was prepared from silkworm cocoons (Soochow University, Soochow, China) using an Na_2_CO_3_/LiBr solution (Sigma-Aldrich, St. Louis, MO, USA). Aqueous SF solutions within a concentration range of 0.5%–2% self-assemble into nanofibers with high β-sheet content, exhibiting tunable mechanical properties and structural features (Bai et al., 2014). Therefore, we prepared 0.5%, 1%, and 2% SF hydrogel solutions to generate hydrogels to be loaded with hNSCs. As shown in **Figure 1A**, a low direct current voltage (50 V) was applied, causing the nanofibers to form a visible hydrogel (E-N-Gel) that expanded from the positive to the negative electrode. Stable gel structures formed after 20, 30, and 40 minutes. The hydrogels were then sliced into 0.5- to 1-mm-thick sections and sterilized in water. Before cell seeding, the slices were irradiated with ultraviolet light for 30 minutes.

### Hydrogel characterization and scanning electron microscopy

Freeze-dried hydrogels were sputter-coated with gold, and a minimum of 300 randomly selected areas per group were analyzed using a Hitachi S-4800 scanning electron microscopy system (3 kV; Hitachi, Tokyo, Japan) and ImageJ Pro Plus software (v6.0, Media Cybernetics, Rockville, MD, USA). The alignment properties of the hydrogels were determined by fast Fourier transform analysis.

### Human-derived neural stem cells isolation and culture

The study protocol for hNSC isolation was approved by the Ethics Committee of the First Affiliated Hospital of Dalian Medical University [No. PJ-KS-KY-2021-128(X); July 16, 2021]. The hNSCs used in this study were extracted and derived from the cerebral cortex of a 14-week-old human fetus. Informed consent for the use of fetal tissue was obtained from the donor’s legal guardian, and all procedures were conducted in accordance with the ethical guidelines set out by the Institutional Ethics Committee. The cells were cultured and maintained in serum-free complete growth medium (HUXNF-90011, Cyagen, Suzhou, China) containing 1x B27 supplement, heparin (10 U/mL), L-glutamine (2 mM), penicillin-streptomycin solution (30 μg/mL), epidermal growth factor (20 ng/mL), and fibroblast growth factor-2 (20 ng/mL). The cells were passaged every 7–10 days using Accutase (A6964, Sigma-Aldrich) and added to new flasks at a density of 2 × 10^5^ cells/mL. When the neurosphere diameter ranged from 80 to 150 µm, the cells were digested and seeded into 12-well plates containing the sterilized hydrogels at a density of 1000 cells/mm^3^. All experiments were performed using cells from passages 5–15.

### hNSC identification and differentiation

After 10 days, the hNSCs were fixed, permeabilized, and verified by immunofluorescence staining using a Human Neural Progenitor Cell Marker Antibody Panel (SC025, R&D Systems, Minneapolis, MN, USA) that detected the following markers: C–X–C motif chemokine receptor 4 (CXCR4), Musashi-1, Nestin, Notch-1, SRY-box transcription factor 1 (SOX1), SRY-box transcription factor 2 (SOX2), stage-specific embryonic antigen-1 (SSEA-1), and Vimentin. Immunofluorescence staining was performed at room temperature for 1 hour according to the panel manufacturer’s instructions, using the appropriate fluorescent secondary antibodies, including goat anti-mouse IgG H&L (TRITC; 1:2000; Abcam, Cambridge, UK; Cat# ab6786; RRID: AB_955514), goat IgG NorthernLights^TM^ NL557-conjugated antibody (1:200, R&D Systems, Cat# NL001, RRID: AB_663766), rat IgG NorthernLights^TM^ NL557-conjugated antibody (1:200, R&D Systems, Cat# NL013, RRID: AB_884217), and mouse IgM NorthernLights^TM^ NL557-conjugated antibody (1:200, R&D Systems, Cat# NL019, RRID: AB_10891132).

Cell differentiation was performed using a NeuroCul NS-A Differentiation Kit (Human) (STEMCELL Technologies, Vancouver, BC, Canada), which contains a standardized medium for differentiating human neural stem and progenitor cells into neurons, astrocytes, and oligodendrocytes. Half of the medium was replaced every 2 days. NSC differentiation efficiency was evaluated using a human/mouse/rat neural three-color immunocytochemistry kit (SC024, R&D Systems) according to the manufacturer’s instructions. The fluorochrome-conjugated antibodies supplied with the kit included anti-oligodendrocyte marker O4 NL557-conjugated mouse IgM, anti-βIII TUBULIN NL637-conjugated mouse IgG2A, and anti-glial fibrillary acidic protein (GFAP) NL493-conjugated sheep IgG. The cells were stained at room temperature for 1 hour.

Fluorescence images were captured using a confocal laser scanning microscope (SP8, Leica, Wetzlar, Germany).

### Cell viability

hNSC viability was assessed using a cell counting kit-8 (CCK-8) kit (DOJINDO Laboratories, Tokyo, Japan). Fifth-, tenth-, and fifteenth-passage hNSCs were seeded into 96-well plates at a density of 5000 cells per well and incubated at 37°C with 5% CO_2_. Next, 10 µL of CCK-8 reagent was added to each well according to the manufacturer’s protocol, the plates were incubated for 2 hours, and the absorbance at 450 nm was measured using a microplate reader (EL808, BioTek, Seattle, WA, USA). The CCK-8 assay was performed for each group of cells every 2 days until day 12, and each condition was tested in triplicate.

### Differentiated cell and synapse morphology

Differentiated cell morphology in the hydrogels was visualized using Calcein AM cell staining reagent (DOJINDO Laboratories) in accordance with the manufacturer’s protocol; live cells emitted green fluorescence (excitation = 490 nm, emission = 515 nm). Synapses were visualized by staining with an antibody against a synapse-specific marker (Anti-Bassoon, 1:1000, Abcam, Cat# ab82958, RRID: AB_1860018) at 4°C for 2 hours, followed by Goat Anti-Mouse IgG2a heavy chain (FITC; 1:100, Abcam, Cat# ab97244, RRID: AB_10680048). Cell nuclei were stained with Hoechst 33258 (Sigma-Aldrich). Fluorescence was observed under a Leica SP8 microscope.

### Quantitative polymerase chain reaction

Differentiated cells were harvested, and total RNA was extracted using TaKaRa MiniBEST Universal RNA Extraction Kit in accordance with the manufacturer’s instructions (Takara Bio, Shiga, Japan). The RNA was reverse-transcribed into complementary DNA using PrimeScript^TM^ RT reagent Kit (Takara). Quantitative polymerase chain reaction was conducted with specific primers for synapse-related genes (**Table 1**) using a fluorescence-based detection system (Bio-Rad Laboratories, Hercules, CA, USA). Relative gene levels were calculated using the ΔΔCt method (Livak and Schmittgen, 2001), with glyceraldehyde-3-phosphate dehydrogenase (*GAPDH*) serving as an internal control.

**Table 1:** PCR primer sequence

Gene	Forward primer (5'–3')	Reverse primer (5'–3')
*NCAM1*	AAT TTA CCG CGG CAA GAA CAT C	CCT GGC TGG GAA CAA TAT CCA C
*SYP*	GCA ATG CCT GCC TGA ACA A	CAC CTT GGG TCC TAA ACT GTC CTC
*PSD95*	AGG ACA TTC AGG CGC ACA AG	CAT TGG CCG AGA CAT CGA G
*GAP43*	GCA GTT CGA CCT AGT CCT TAT TCA C	TTG CGG CCT TAT GAG CTT TAT C
*GAPDH*	GCA CCG TCA AGG CTG AGA AC	TGG TGA AGA CGC CAG TGG A

GAP43: Growth-associated protein 43; GAPDH: glyceraldehyde-3-phosphate dehydrogenase; NCAM1: neural cell adhesion molecule 1; PCR: polymerase chain reaction; PSD95: postsynaptic density protein-95; SYP: synaptophysin.

### Hypoxic-ischemic encephalopathy rat model establishment and human-derived neural stem cell treatment

All animal procedures were approved by the Animal Experimental Ethical Inspection Committee of Dalian Medical University (No. AEE22056; March 29, 2024). Unilateral carotid artery ligation followed by hypoxia in 7-day-old postnatal rats induces selective neuronal death in the ipsilateral cortex, striatum, hippocampus, as well as white matter necrosis, providing valuable insights into HIE pathophysiology (Vannucci and Vannucci, 2005). To establish this model, pregnant Sprague–Dawley rats (gestational days 16 and 17) were obtained from the specific pathogen-free Experimental Animal Center of Dalian Medical University (Dalian, China; license No. SCXK2018-0003) and housed individually in standard plastic cages with free access to food and water, a controlled temperature, and a 12/12-hour light/dark cycle. After delivery, 7-day-old hairless juvenile rats (weighing 20 ± 3 g; male:female = 1:1, *n* = 10 rats/group) were subjected to HIE. Briefly, under isoflurane (R510-22, RWD, Shenzhen, China) anesthesia (4% induction and 2% maintenance), the right common carotid artery was permanently ligated through a midline neck incision. The surgical wound was closed with sutures immediately after ligation. Room temperature was maintained at 24–25°C during surgery. Subsequently, the juvenile rats were returned to their mothers for a 2-hour recovery. Next, they were transferred to a hypoxia chamber maintained at 8% oxygen for 2 hours then returned to normoxic condition. Once normal tail reflexes were observed, the juvenile rats were returned to maternal care.

On day 10 after birth (3 days post-injury, P3), the rats were randomly assigned to three groups: HIE, hNSC treatment (NSC), and hNSC combined with ordered SF hydrogel treatment (NSC + silk). The rats were weighed, anesthetized with 4% isoflurane, and placed in a stereotaxic apparatus. A 0.5-cm midline incision was made to expose the skull. In the NSC group, rats received a single deposit of cell suspension (1 × 10^5^ cells, 10 μL) into the peri-infarct cortex region at a depth of approximately 5 mm using a stereotaxic-guided microsyringe (68001, RWD), as previously described (Smith et al., 2012). In the NSC + silk group, 1 × 10^5^ NSCs were seeded onto a silk protein hydrogel that was placed directly at the injury site using forceps, as previously described (Kim et al., 2023). Rats in the HIE group were injected with 10 μL of saline at the brain injury site. Finally, the scalp was sutured. Behavioral experiments (cylinder, balance beam, and water maze tests) were conducted starting on 21 days post-injury (P21). Brain samples were collected on 32 days post-injury (P32) under deep anesthesia (10% isoflurane), fixed in 4% paraformaldehyde, paraffin-embedded, and sectioned into 3-μm-thick slices for hematoxylin and eosin (HE) staining, GFAP immunohistochemistry, and immunofluorescence staining for GFAP, Tubulin-3, and NeuN.

### Rat behavior tests

Balance beam and cylinder tests were used to evaluate limb impairment and motor coordination (Brooks and Dunnett, 2009), while the Morris water maze test was used to assess spatial learning and cognitive function (Boboc et al., 2023).

#### Balance beam test

The balance beam (Shanghai Newsoft, Shanghai, China) test employed a standard square arena and round horizontal beam (1 m × 5 cm × 5 cm) placed at a height of 50 cm. Prior to the test, each rat underwent three consecutive training sessions. The average score was calculated from three trials. The scoring criteria are presented in **Additional Table 1**.

**Additional Table 1:** Scoring criteria for animal balance beam test

Score	Parameter	Time
1	Excellent balance, no sign of wobbling	> 2 min
2	Slight wobbling, quickly corrects balance	> 2 min
3	Moderate wobbling, unilateral slip but no fall off beam	> 2 min
4	Substantial wobbling, struggles to maintain balance but falls off beam	< 2 min
5	Severe wobbling, falls off beam	Seconds
6	No standing ability	-

#### Cylinder test

The rats were allowed to adapt to the area for 30 minutes undisturbed at a comfortable ambient temperature and humidity. Each rat was then held by its tail and placed into a transparent glass cylinder (radius = 10 cm, height = 30 cm). Video monitoring software (XR-Xmaze^+^, Shanghai Xinruan Information Technology, Shanghai, China) was used to record the number of times the rat touched the cylinder wall with the contralateral forelimb within 5 minutes. After testing each rat, the cylinder was disinfected with 75% alcohol, dried, and prepared for the next rat. Each rat underwent three tests. The percentage of touches on the left (impaired) side was calculated as the number of impaired-side touches/total number of touches × 100.

#### Morris water maze test

The Morris water maze test was used to evaluate spatial learning and memory abilities. As described in a previous study (Boboc et al., 2023), the pool (Shanghai Xinruan Information Technology) was divided into four equal quadrants, with a platform (diameter = 10 cm, height = 30 cm) located in the center of the fourth quadrant. Tests started from the 28^th^ day after birth (P21); test days 1–4 involved the place navigation trial and day 5 involved the spatial probe trial. Warm water (22–24°C) was added to the arena to a level 1 cm above the platform. During the first 4 days, each rat was placed in the water at a random location with its backs to the pool wall. The time required (escape latency) and route followed (escape distance) to locate the platform were recorded for each rat. If a rat did not find the platform within 90 seconds, it was manually guided to it and allowed to stay on it for 10 seconds before being removed from the pool and dried. On day 5, the platform was removed from the pool, each rat was placed in the opposite quadrant (second quadrant) to the platform’s previous location and allowed to swim for 1 minute, and its path was tracked. The total time spent swimming in the fourth quadrant (crossing target quadrant time) and the number of times the rat crossed the center of fourth quadrant (crossing platform times) were recorded. The swimming paths and data were analyzed using tracking software (Shanghai Xinruan Information Technology).

### Immunohistochemistry

The rats were euthanized by cervical dislocation under deep anesthesia (10% isoflurane; RWD). Brains were immediately removed, fixed in 4% paraformaldehyde (24 hours, 4°C), and processed into paraffin blocks. Coronal sections (2 μm) were cut using a microtome (Leica RM2235) and mounted on glass slides. Sections were deparaffinized in xylene (Sigma-Aldrich; 3 × 5 minutes), rehydrated through ethanol concentrations (100%–70%), and antigen-retrieved in citrate buffer (pH 6.0) at 95°C for 20 minutes. Endogenous peroxidase was blocked with 3% H_2_O_2_/methanol (Thermo Fisher, H325; 10 minutes, room temperature), followed by incubation with a sheep anti-rat GFAP polyclonal primary antibody (10 μg/mL, R&D Systems, Cat# AF2594; RRID: AB_2109656) for 3 hours at room temperature. The sections were then incubated with rabbit anti-sheep IgG H&L (1:1000, Abcam, Cat# ab6747; RRID: AB_955453) for 1 hour at room temperature, followed by treatment with chromogenic DAB liquid substrate to visualize antibody binding (Abcam). The sections were analyzed under a Leica SP8 microscope.

### Immunofluorescence staining

Cells and brain slices were fixed with 4% paraformaldehyde, permeabilized, and blocked to prevent nonspecific binding (Beyotime, Haimen, China). They were then incubated overnight at 4°C with the following primary antibodies: rabbit anti-rat GFAP polyclonal antibody (1:5000, Abcam, Cat# ab7260, RRID: AB_305808), rabbit anti-rat Tubulin-3 polyclonal antibody (1:1000, Abcam, Cat# ab18207, RRID: AB_444319), and rabbit anti-rat NeuN monoclonal antibody (1:200, Abcam, Cat# ab177487, RRID: AB_2532109). Next, they were incubated with the following secondary antibodies for 1 hour at room temperature: Goat Anti-Rabbit IgG H&L (HRP; 1:2000, Abcam, Cat# ab7090, RRID: AB_955417) and Goat Anti-Rabbit IgG H&L (1:1000, Alexa Fluor® 488; Abcam, Cat# ab150081; RRID: AB_2734747). The samples were then stained with Hoechst 33258 (Sigma-Aldrich) to visualize the nuclei and mounted for imaging. Fluorescence was observed under a Leica SP8 microscope.

### Enzyme-linked immunosorbent assay

Another 150 juvenile rats (P7, weighing 20 ± 3 g) were selected and divided into HIE, NSC, and NSC + silk groups (*n* = 50 rats/group) that were subjected to the same protocols discussed above. On the 8^th^ (pre-treatment), 11^th^ (day 1 post-treatment), 13^th^ (day 3 post-treatment), 17^th^ (day 7 post-treatment), and 39^th^ (day 29 post-treatment) day after birth, 10 rats per group were euthanized by cervical dislocation under deep anesthesia with 10% isoflurane. The cortex was carefully separated to access the hippocampal tissue, which was then removed, rinsed with ice-cold phosphate saline, and stored in sterile Eppendorf tubes at –80°C. Hippocampal lysates and SF solution supernatants (from various concentrations) were analyzed for BDNF (DBNT00, R&D Systems) and NGF (EHNGF, Thermo Fisher) levels using ELISA kits per manufacturer protocols.

### Statistical analysis

The evaluator was blinded to the group assignments to minimize bias during data collection and analysis. Statistical analyses were conducted using SPSS 22.0 software (IBM SPSS, Armonk, NY, USA), and GraphPad Prism software (version 9.0.0, GraphPad Software, San Diego, CA, USA, www.graphpad.com) was used for data visualization. Data are presented as the mean ± standard deviation (SD). A two-sample *t*-test was performed to compare means between two groups, while one-way analysis of variance followed by least significant difference *post hoc* test (homogeneity of variances) or Welch test with Dunnett *post hoc* test (heterogeneity of variances) was employed for comparisons among multiple groups. For repeated measures data collected at different time points, repeated measures analysis of variance with Sidak *post hoc* test was applied. Statistical significance was set at *P* < 0.05.

## Results

### Electric field enables directional nanofiber alignment and stratification in silk fibroin hydrogels

Under a 50 V electric field, negatively charged SF molecules clustered at the positive pole, forming intertwining nanofibers with a nano-oriented structure (**Figure 1A**). Electrostatic repulsion stratified these negatively charged SF molecules. High-magnification electron microscopy revealed the laminated orientations of the nanostructures within the layers. SF hydrogels not subjected to electric field treatment exhibited a disordered nanofiber arrangement (**Figure 1B**). By contrast, electric field treatment induced the formation of aligned SF nanofiber layers (**Figure 1C**). These findings underscore the impact of electric fields on the organization of nanofibers in SF hydrogels.

### Primary culture and characterization of human-derived neural stem cells

**Figure 2:**
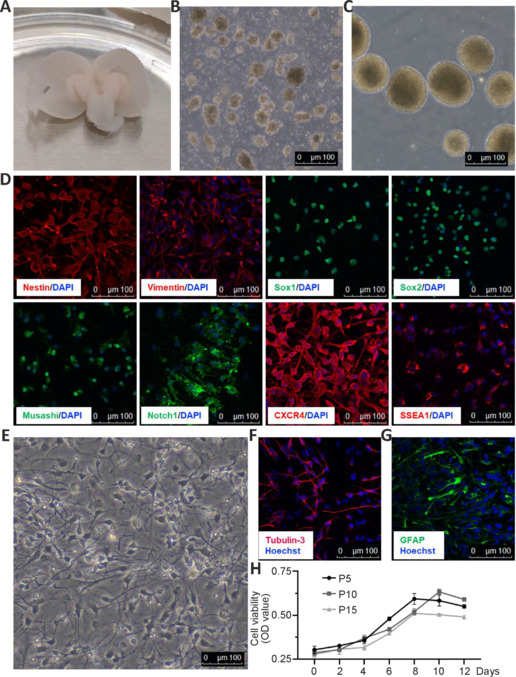
Characterization of hNSCs. (A) Whole brain tissue was isolated from an aborted embryo at 14 weeks of gestation. (B) Morphology of subventricular-zone cells derived from fetal tissue after 9 days of *in vitro* culture, as assessed by light microscopy (40× original magnification; scale bar: 100 μm). (C) Light microscopy images of neurospheres displaying microspikes along the periphery; on the 14^th^ day of culture, they developed dark cores, became overgrown, and began attaching to the dish surface, potentially indicating spontaneous differentiation (40× original magnification; scale bar: 100 μm). (D) Immunofluorescence staining of cultured hNSCs for Nestin-TRITC (red), Vimentin-TRITC (red), SOX1-NL557 (green), SOX2-NL557 (green), Musashi-1-NL557 (green), Notch-1-NL557 (green), CXCR4-TRITC (red), and SSEA-1-TRITC (red), with nuclei counterstained using DAPI (blue) (scale bar: 100 µm). (E) Morphological changes in hNSCs that differentiated into neural cells after 18 days (40× original magnification; scale bar: 100 μm). (F) Tubulin-3-NL637 (red) staining indicating neuronal differentiation, with nuclei counterstained using Hoechst 33258 (blue) (scale bar: 100 µm). (G) GFAP-NL493 (green) staining indicating astrocytic differentiation, with nuclei counterstained using Hoechst 33258 (blue) (scale bar: 100 µm). (H) Proliferation curve of 5^th^-, 10^th^-, and 15^th^-generation (P5, P10, and P15) hNSCs, as determined by CCK-8 assay (*n* = 3/group). Data are expressed as mean ± SD and were analyzed by repeated measures analysis of variance. CCK-8: Cell counting kit-8; CXCR4: C–X–C motif chemokine receptor 4; DAPI: 4′,6-diamidino-2-phenylindole; GFAP: glial fibrillary acidic protein; hNSC: human-derived neural stem cell; OD: optical density SOX1: SRY-box transcription factor 1; SOX2: SRY-box transcription factor 2; SSEA-1: stage-specific embryonic antigen-1.

Brain tissue samples, specifically from the subventricular zone, were obtained from an aborted embryo at 14 weeks of gestation (**Figure 2A**), mechanically minced, and cultured in hNSC culture medium. After approximately 7–10 days of primary culture, P0 neurospheres of various shapes and sizes had formed (**Figure 2B**). The subsequent passage (P1) showed a more purified population of NSC spheres (**Figure 2C**). The neurospheres were characterized by a distinct inner boundary, strong refractive properties, and minimal amounts of dead cells. Dark cores, overgrowth, or adherence and differentiation typically appeared every 4–7 days, indicating that passaging was necessary.

We identified hNSCs on the basis of their expression of characteristic biomarkers and differentiation potential. The primary hNSCs expressed various neural progenitor cell surface markers, as previously described (Tennstaedt et al., 2015; **Figure 2D**). We assessed the multipotency of the hNSCs by evaluating their ability to differentiate into neurons, astrocytes, and oligodendrocytes, as indicated by the expression of specific markers (Tubulin-3, GFAP, and O4, respectively) (Santos et al., 2022). hNSCs were cultured in differentiation medium. After 10–18 days, the differentiated cells formed an interconnected network of dense processes (**Figure 2E**) and expressed Tubulin-3 (**Figure 2F**) and GFAP (**Figure 2G**). These results confirmed that the extracted cells were hNSCs and suitable for use in subsequent experiments.

We performed a CCK-8 assay to evaluate cell proliferation and observed slow growth from days 0–4 post-passage, followed by a rapid growth phase. By day 12, most cells had reached a diameter of 100 μm, requiring passaging. No significant differences were observed among the P5, P10, and P15 groups (**Figure 2H**). These findings suggest that hNSCs are capable of sustained proliferation, and that passage number may not significantly influence cell growth dynamics within the assessed range.

### 1% silk fibroin hydrogel optimally supports human-derived neural stem cell growth, neuriteextension, and neurotrophic factor secretion

**Figure 3:**
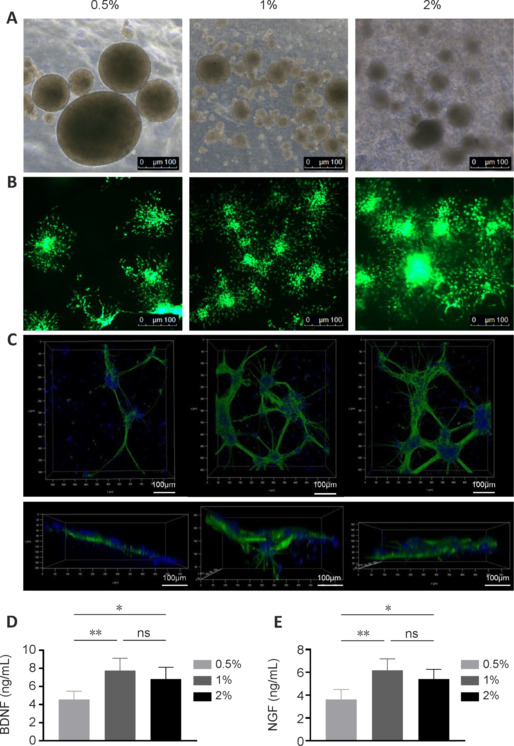
hNSC behavior in different SF hydrogel concentrations. (A) Neural spheres generated in three different concentrations of SF hydrogels (40× original magnification; scale bars: 100 μm). After 7 days of culture, hNSCs in 0.5% and 1% SF hydrogels formed uniform neurospheres with clear boundaries and minimal necrosis, while those in 2% SF hydrogel showed unclear boundaries and were embedded in material voids. (B) hNSCs differentiated on SF hydrogels of different concentrations on day 7, with extended neurites visualized by Calcein-AM staining (green) (scale bars: 100 µm). (C) Differentiated cell axons elongated and formed two-dimensional connections (top) and three-dimensional structures (bottom) on day 7, as visualized by Calcein-AM staining (green), with nuclei counterstained using Hoechst 33258 (blue) (scale bars: 100 µm). Axon growth was enhanced by growth on 1% and 2% SF hydrogels, with 1% SF promoting greater connection and extension. (D, E) Concentrations of BDNF and NGF produced by hNSCs grown in SF hydrogels of different concentrations. Data are presented as the mean ± SD (*n* = 5/group). *P < 0.05, **P < 0.01 (one-way analysis of variance followed by the least significant difference *post hoc* test). BDNF: Brain-derived neurotrophic factor; hNSC: human-derived neural stem cell; NGF: nerve growth factor; SF: silk fibroin.

To determine the optimal hydrogel concentration for hNSC growth, equal numbers of hNSCs were cultured on ordered hydrogel slices produced using varying concentrations of SF. As shown in **Figure 3A**, after 7 days of culture, hNSCs in the 0.5% and 1% SF hydrogels exhibited healthy growth, with uniformly sized spheres that exhibited minimal central necrosis, while some neural spheres in the 2% SF hydrogel became embedded within the material voids. Additionally, hNSCs extended neurites on the SF hydrogels (**Figure 3B**), with more pronounced neurite growth on the 1% and 2% SF hydrogels than on the 0.5% SF hydrogel (**Figure 3C**). The 1% SF hydrogel particularly promoted neurite connection and longitudinal extension (**Figure 3C**).

Neurotrophic factors are crucial for promoting neuronal differentiation and synapse formation in seeded NSCs (Sun et al., 2020). To clarify the reasons for the observed differences in neurite formation on hydrogels with varying SF concentrations, we assessed the levels of BDNF and NGF in the culture medium. As shown in **Figure 3D** and **E**, the concentrations of BDNF and NGF were higher in the 1% and 2% SF hydrogels than in the 0.5% SF hydrogel. However, no significant difference in BDNF or NGF levels was observed between the 2% and 1% SF hydrogels, suggesting that the neurotrophic factors in the culture medium were secreted by the hNSCs rather than by the SF hydrogels.

Collectively, these findings confirm that SF hydrogel supports hNSC growth and neurotrophic factor secretion and indicate that a 1% SF hydrogel concentration is optimal for promoting axon elongation. Thus, this concentration was used in subsequent experiments.

### Ordered silk fibroin hydrogels enhance neuronal differentiation and axon elongation in human-derived neural stem cell

**Figure 4:**
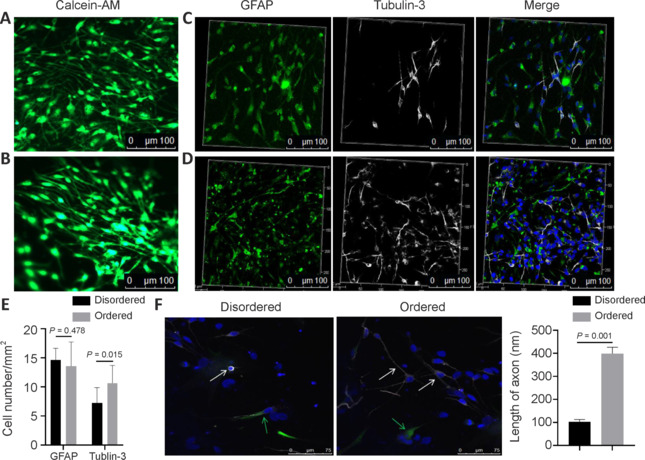
Immunofluorescence analysis of hNSC differentiation on SF hydrogels. (A, B) hNSCs differentiated on disordered (A) and ordered (B) SF hydrogels, as visualized by Calcein-AM staining (scale bar: 100 µm), with no significant differences observed between the two groups. (C, D) Representative immunofluorescence images of GFAP-NL493 (green, marking astrocytes) and Tubulin-3-NL637 (gray, marking neurons) staining of cells cultured on disordered (C) and ordered (D) SF hydrogels. Nuclei were stained with Hoechst 33258 (blue) (scale bars: 100 µm). The number of Tubulin-3-positive neurons was significantly increased in the ordered SF hydrogel group compared with the disordered hydrogel group, whereas the number of GFAP-positive astrocytes showed no significant difference between the groups. (E) Quantification of astrocytes and neurons (*n* = 10/group). (F) Axon length analysis (*n* = 30/group). Neuronal axons of hNSCs grown on disordered and ordered SF hydrogels were stained with Tubulin-3-NL637 (gray), astrocytes with GFAP-NL493 (green), and nuclei with Hoechst 33258 (blue) (scale bars: 75 µm). Differentiated neurons displayed significantly longer axons on ordered SF hydrogels compared with disordered SF hydrogels. The gray arrows point to neurons, and the green arrows point to astrocytes. Data are presented as the mean ± SD. *P < 0.05 (two-sample *t*-test). GFAP: Glial fibrillary acidic protein; hNSC: human-derived neural stem cell; SF: silk fibroin.

To clarify the effect of SF fiber arrangement on hNSC differentiation direction, we analyzed the proportion of neurons and astrocytes in differentiated hNSCs cultured on disordered and ordered SF hydrogels. After 14 days of induced differentiation, hNSCs in both groups exhibited axon elongation. However, compared with those on the disordered hydrogel (**Figure 4A**), differentiated cells on the ordered hydrogel showed more regular growth and extension, resulting in the formation of a well-organized neural network (**Figure 4B**). Immunofluorescence staining revealed the presence of neurons and astrocytes on both disordered (**Figure 4C**) and ordered (**Figure 4D**) SF hydrogels. Statistical analysis showed that the number of differentiated neurons (Tubulin-3) was significantly higher (*P* = 0.015) in the ordered group (10.7 ± 3.0 cells/mm^2^) than in the disordered group (7.2 ± 2.7 cells/mm^2^, **Figure 4E**). No significant difference was observed in astrocyte counts (*P* = 0.478, ordered: 13.6 ± 4.1 cells/mm^2^, disordered: 14.6 ± 2.0 cells/mm^2^). Cells cultured on the ordered hydrogels (399.42 ± 26.90 nm) exhibited significantly longer axons (*P* < 0.001) than those cultured on the disordered hydrogels (103.76 ± 9.48 nm) (**Figure 4F**).

These findings demonstrate that ordered SF hydrogels promote hNSC differentiation into neurons, enhance directional axon extension, and support neural network formation, suggesting that the structural characteristics of SF hydrogels play a critical role in neuronal development.

### Ordered silk fibroin hydrogels enhance synaptic protein expression and functional maturation in differentiated neurons

**Figure 5:**
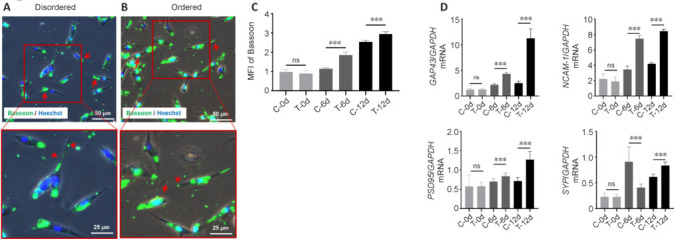
Synapse analysis of differentiated neurons cultured on SF hydrogels. (A, B) Representative immunofluorescence staining images of Bassoon–FITC (green, synapse-specific marker) after 14 days of culturing on disordered (A) and ordered (B) SF hydrogels; nuclei were stained with Hoechst 33258 (blue). Immunofluorescence analysis on day 12 showed that hNSCs differentiated on both disordered and ordered SF hydrogels expressed Bassoon. The red arrows indicate Bassoon immunoreactivity. (C) Mean fluorescence intensity (MFI) of Bassoon in differentiated neurons cultured on ordered and disordered SF hydrogels. (D) *GAP43*, *NCAM-1*, *PSD95*, and *SYP* mRNA levels in differentiated cells cultured on ordered and disordered SF hydrogels. Number of days required for hNSC differentiation in the C-nd group (hNSCs cultured on disordered SF hydrogels) and T-nd group (hNSCs cultured on ordered SF hydrogels). Data are presented as the mean ± SD (*n* = 10/group). ***P < 0.001 (two-sample *t*-test). GAP43: Growth-associated protein 43; GAPDH: glyceraldehyde-3-phosphate dehydrogenase; hNSC: human-derived neural stem cell; NCAM1: neural cell adhesion molecule 1; ns: not significant; PSD95: postsynaptic density protein-95; SF: silk fibroin; SYP: synaptophysin.

To further clarify the effect of ordered SF hydrogels on the function of differentiated neurons, we investigated the synaptic structure of the expression of synaptic function-related proteins by neurons differentiated on ordered and disordered SF hydrogels after 0, 6, and 12 days of culturing. Bassoon protein, a key component of the presynaptic plasma membrane, is essential for synapse assembly and structural maintenance, organizing presynaptic release sites, aggregating synaptic vesicles, and facilitating efficient neurotransmitter release (Waites et al., 2013). Immunofluorescence staining revealed that, by day 12, hNSCs differentiated on both disordered (**Figure 5A**) and ordered (**Figure 5B**) SF hydrogels expressed Bassoon protein. Continuous immunofluorescence analysis indicated that, from day 6, Bassoon protein expression was higher in cells on ordered SF hydrogels than those differentiated on disordered SF hydrogels (*P* < 0.001; **Figure 5C**). Further analysis of synapse-related genes showed that the mRNA levels of *GAP43*, *NCAM1*, and *PSD65* were higher in differentiated hNSCs cultured on ordered hydrogels than on disordered hydrogels on days 6 and 12 (*P* < 0.001; **Figure 5D**). Additionally, *SYP* expression decreased on day 6 but significantly increased by day 12 (*P* < 0.001; **Figure 5D**). These results indicate that ordered SF hydrogels enhance synaptic structure and functional protein expression in differentiated neurons, highlighting their beneficial role in promoting neuronal functionality.

### Neural stem cells combined with silk reduces infarction and reactive gliosis while enhancing neuroprotection in hypoxic-ischemic encephalopathy rats

**Figure 6:**
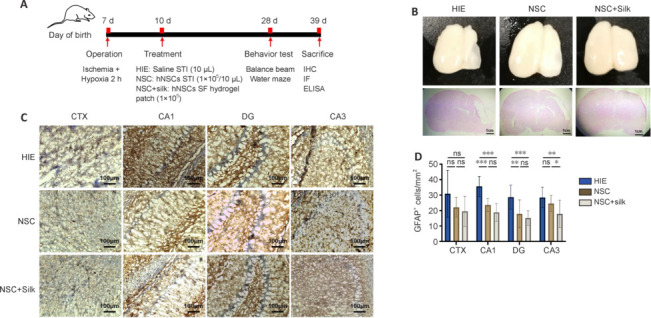
Efficacy assessments in the HIE rat model. (A) Animal experiment timeline. (B) Whole-brain and HE-staining images. The infarct area was significantly reduced in both the hNSC direct injection group and the hNSC-loaded SF hydrogel group compared with the HIE group. Scale bars: 1 cm. (C) Representative IHC images of GFAP in the cerebral cortex and hippocampal CA1, DG, and CA3 regions (scale bars: 100 μm). (D) Number of GFAP-positive cells. Data are presented as the mean ± SD (*n* = 10/group). *P < 0.05, **P < 0.01, ***P < 0.001 (one-way analysis of variance followed by the least significant difference *post hoc* test or Welch test with Dunnett *post hoc* test). CTX: Cerebral cortex; DG: dentate gyrus; ELISA: enzyme-linked immunosorbent assay; GFAP: glial fibrillary acidic protein; HE staining: hematoxylin–eosin staining; HIE: hypoxic-ischemic encephalopathy; hNSC: human-derived neural stem cell; IF: immunofluorescence; IHC: immunohistochemistry; ns: not significant; NSC: neural stem cell; SF: silk fibroin; STI: stereotaxic injection.

The above findings indicated that a 1% ordered silk hydrogel promoted hNSC proliferation, neurotrophic factor secretion, neuronal differentiation, axonal growth, and the establishment of functional synapses ex vivo. Next, we evaluated the therapeutic effect of hNSCs alone (stereotaxic injection) and hNSCs loaded into ordered SF hydrogel scaffolds (implantation) *in vivo* in 7-day-old rats (**Figure 6A**) subjected to HIE by right common carotid artery dissection followed by 2 hours of hypoxia (8% oxygen concentration). Whole-brain imaging and HE staining revealed infarction in the hippocampus, cortex, and striatum (**Figure 6B**). However, the infarct area was significantly reduced following hNSC treatment, indicating that both direct injection of hNSCs and implantation of the hNSC-loaded SF hydrogel repaired the damage caused by cerebral infarction.

Under ischemic conditions, astrocytes undergo a characteristic transformation known as reactive astrogliosis (or astrocyte activation) marked by GFAP upregulation and the formation of a glial scar that hinders nerve repair during the chronic phase post-ischemia (Hol and Pekny, 2015; He et al., 2022). Immunohistochemistry analysis revealed a significant decrease in the number of GFAP^+^ cells in the hippocampal CA1 (NSC *vs*. HIE, *P* < 0.001; NSC + silk *vs.* HIE, *P* < 0.001) and dentate gyrus (NSC *vs*. HIE, *P* = 0.003; NSC + silk *vs*. HIE, *P* < 0.001) regions 29 days after treatment, regardless of the method of hNSC administration, compared with the HIE control group (**Figure 6C** and **D**). No change was observed in the cerebral cortex (*P* > 0.05). Specifically, the number of GFAP^+^ cells in the hippocampal CA3 region was lower in the NSC + silk group than in the NSC group (NSC + silk *vs.* NSC, *P* = 0.044; **Figure 6D**).

Collectively, these results indicate that both direct injection of hNSCs and implantation of hNSC-loaded ordered SF hydrogel scaffolds promote functional recovery in 7-day-old HIE rats by reducing cerebral infarction, decreasing reactive astrocyte activation in the hippocampal region, and enhancing neuroprotection, and that the strongest effects were seen in the CA3 region when the SF hydrogel was applied.

### Human-derived neural stem cells promote neuronal regeneration in hypoxic-ischemic encephalopathy rats

**Figure 7:**
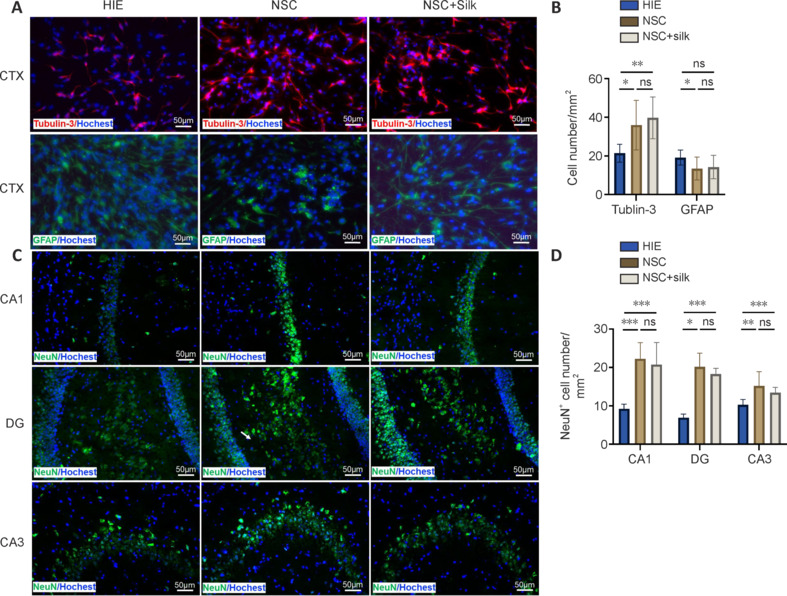
Immunofluorescence analysis of the cerebral cortex and hippocampus in HIE rats. (A) Representative immunofluorescence images of cerebral cortex sections showing neurons (Tubulin-3-NL637, red) and astrocytes (GFAP-NL493, green). Nuclei were stained with Hoechst 33258 (blue) (scale bars: 50 µm). (B) Number of Tubulin-3- and GFAP-positive cells in the cerebral cortex. (C) Representative immunofluorescence images of sections from different regions of the hippocampus showing neurons (NeuN-Alexa Fluor® 488, green). Nuclei were stained with Hoechst 33258 (blue) (scale bars: 50 µm). (D) Number of NeuN-positive cells in different hippocampal regions. Data are presented as the mean ± SD (*n* = 10/group). *P < 0.05, **P < 0.01, ***P < 0.001 (one-way analysis of variance followed by the least significant difference *post hoc* test (B) or Welch test with Dunnett *post hoc* test (D)). GFAP: Glial fibrillary acidic protein; HIE: hypoxic-ischemic encephalopathy; ns: not significant; NSC: neural stem cell.

To determine whether hNSCs and hNSC-loaded SF hydrogel promote neuronal regeneration *in vivo*, we analyzed neurons (marked by Tubulin-3 and NeuN) and astrocytes (marked by GFAP) in different brain regions after HIE. Compared with the HIE injury group, both the NSC and NSC + silk treatment groups exhibited significantly increased Tubulin-3 expression in the cerebral cortex (**Figure 7A**). Quantitative analysis of cortical cells revealed a higher number of Tubulin-3^+^ cells in the NSC and NSC + silk groups than in the HIE group (NSC *vs*. HIE, *P* = 0.014; NSC + silk *vs*. HIE, *P* = 0.001, **Figure 7B**), with no significant difference between the NSC and NSC + silk groups (*P* = 0.900; **Figure 7B**). GFAP expression (**Figure 7A**) and GFAP^+^ cell counts (**Figure 7B**) were decreased in the NSC group (*P* = 0.026) compared with the HIE group, but no significant change was found in the NSC + silk group compared with the HIE group (*P* = 0.052), consistent with the Immunohistochemistry staining results.

Regarding hippocampal regions, the NSC and NSC + silk groups showed higher NeuN levels (**Figure 7C**) and NeuN+ cell counts (**Figure 7D**) in the CA1 (NSC *vs.* HIE, *P* < 0.001; NSC + silk *vs.* HIE, *P* < 0.001), dentate gyrus (NSC *vs.* HIE, *P* < 0.001; NSC + silk *vs.* HIE, *P* < 0.001), and CA3 (NSC *vs*. HIE, *P* = 0.005; NSC + silk *vs.* HIE, *P* < 0.001) regions than the HIE group. There was no difference in hippocampal neuron regeneration rates between the NSC and NSC + silk treatment groups (CA1, *P* = 0.869; dentate gyrus, *P* = 0.370; CA3, *P* = 0.423; **Figure 7D**).

These results indicated that hNSCs promoted neuronal regeneration in both the cerebral cortex and the hippocampus following HIE injury, while the addition of ordered SF hydrogel did not provide further improvement.

### Silk fibroin hydrogel enhances human-derived neural stem cell-induced neurotrophic factor release

**Figure 8:**
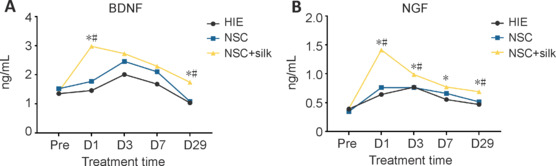
Neurotrophic factor release in the hippocampus of HIE rats induced by implantation of hNSCs and an SF hydrogel. (A, B) ELISA analysis of BDNF (A) and NGF (B) levels in rat hippocampal tissue. Data are presented as mean values (*n* = 10/group). *P < 0.05, *vs*. HIE group; #*P* < 0.05, *vs.* NSC groups (repeated-measures analysis of variance followed by Sidak *post hoc* test). BDNF: Brain-derived neurotrophic factor; D: day; ELISA: enzyme-linked immunosorbent assay; HIE: hypoxic-ischemic encephalopathy; hNSC: human-derived neural stem cell; NGF: nerve growth factor; NSC: neural stem cell; SF: silk fibroin.

hNSCs ameliorated HIE injury by reducing cerebral lesions and astrocyte reactivity, as well as promoting neural regeneration in the cerebral cortex and hippocampus; however, no additional advantages were observed when the hNSCs were loaded into ordered SF hydrogels. Neurotrophins have been shown to exert cytoprotective, anti-inflammatory, and angiogenic effects in rats with ischemic stroke (Salikhova et al., 2021). To further examine dynamic changes in neurotrophic factor levels in hippocampal tissue during HIE and the effects of different hNSC administration methods, hippocampal tissue was collected before hNSC treatment and 1, 3, 7, and 29 days after treatment. As shown in **Figure 8**, no significant differences in BDNF and NGF levels were observed among the groups before treatment (*P* > 0.05). However, 1 day after treatment, BDNF and NGF levels in the NSC + silk group increased rapidly and were significantly higher than those in the NSC (*P* < 0.001) and HIE- groups (*P* < 0.001). Despite an ongoing decrease in BDNF and NGF levels, NGF levels in the NSC + silk group remained higher than those in the NSC (D1, *P* < 0.001; D3, *P* = 0.002; D7, *P* = 0.070; D29, *P* < 0.001) and HIE (D1, *P* < 0.001; D3, *P* = 0.002; D7, *P* < 0.001; D29, *P* < 0.001) groups at nearly all time points. These findings suggest that, while hNSCs alone can promote repair following HIE, the combination of hNSCs with SF hydrogel induces a rapid and substantial increase in neurotrophic factor levels, supporting the use of SF hydrogel and hNSCs together for more effective and sustained neuroprotection in the context of HIE.

### Silk fibroin hydrogel augments human-derived neural stem cell therapy for cognitive but not motor recovery in hypoxic-ischemic encephalopathy rats

**Figure 9:**
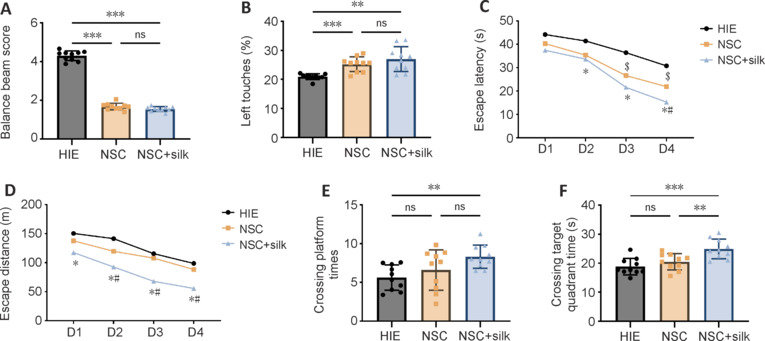
Behavioral performance of HIE rats. (A) Balance beam scores. (B) Cylinder test results. (C–F) Morris water maze escape latency (C), distance (D), platform crossing times (E), and target quadrant crossing time (F). Data are presented as mean values (*n* = 10/group). *P < 0.05, **P < 0.01, ***P ≤ 0.001 (*vs.* HIE group in C and D); #*P* < 0.05, *vs.* NSC groups (Welch test with Dunnett *post hoc* test (A, B, E), repeated-measures analysis of variance followed by Sidak *post hoc* test (C, D), one-way analysis of variance followed by the least significant difference *post hoc* test (F)). HIE: Hypoxic-ischemic encephalopathy; ns: not fignificant; NSC: neural stem cell.

To further evaluate the therapeutic effects of hNSCs and hNSC-loaded SF hydrogel on neurological function in HIE rats, behavioral tests were conducted starting 21 days post-injury (18 days post-treatment). The statistical results are summarized in **Additional Tables 2** and **3**. Ligation and dissection of the right common carotid artery resulted in significant motor function impairment in the left limb. Motor function was improved in the NSC and NSC + silk groups compared with the HIE group, as evidenced by better performance on the balance beam test (*P* < 0.05; **Figure 9A**) and increased left forelimb usage (*P* < 0.05; **Figure 9B**). However, no significant differences were observed between the NSC and NSC + silk treatment groups in either the balance beam or the cylinder test (*P* > 0.05).

**Additional Table 2:** Balance beam scores in HIE rat models.

Group	Balance score	*P*-value
HIE group	4.30 ±0.25	-
NSC group	1.68 ±0.17***	<0.001
NSC + silk group	1.55 ± 0.12***	<0.001

Data are presented as the mean ± SD (*n* = 10/group). ***P < 0.001, *vs*. HIE group (Welch test with Dunnett post hoc test). HIE: Hypoxic-ischemic encephalopathy; NSC: neural stem cell.

**Additional Table 3:** Cylinder test statistics in HIE rat models

Group	Left touches (%)	*P*-value
HIE group	20.96 ± 1.01	-
NSC group	25.25 ±2.53**	0.001
NSC + silk group	27.02 ±4.29**	0.004

Data are presented as the mean ± SD (*n* = 10/group). **P < 0.01, *vs*. HIE group (Welch test with Dunnett post hoc test). HIE: hypoxic-ischemic encephalopathy; NSC: neural stem cell.

Cognitive function was assessed using the Morris water maze test (**Additional Table 4**). There were no differences in spatial orientation abilities among the groups prior to training, as indicated by similar escape latencies and distances (*P* > 0.05; **Figure 9C** and **D**), except for rats in the NSC + silk group exhibiting a shorter escape distance than those in the HIE group, likely due to better motor function 18 days post-treatment (*P* = 0.023). During the 4-day training period, all groups showed reduced escape latencies and distances compared to their respective baseline levels on day 1, with hNSC treatment resulting in shorter escape latencies compared with the control group starting from day 3 (**Figure 9C**). The rats in the NSC + silk group showed more rapid and greater improvements in both escape latency and distance than those in the HIE control and NSC groups (*P* < 0.05; **Figure 9C** and **D**). Notably, on day 5, after the platform removal, the rats in the NSC + silk group exhibited better spatial orientation and memory than those in the HIE and NSC groups, as indicated by a greater number of platform crossings and more time spent in the platform area (*P* < 0.05; **Figure 9E** and **F**).

**Additional Table 4:** Morris water maze test results in HIE rat models.

		HIE	NSC	NSC + silk	*P-value	^#^*P*-value	^$^*P*-value
Escape latency (s)	Day 1	44.17 ±2.32	40.27 ±2.32	37.43 ±2.32	0.141	0.776	0.569
	Day 2	41.42 ± 2.12	35.34 ± 2.12	33.65 ±2.12^*^	0.046	0.926	0.151
	Day 3	36.38 ±1.51	26.61 ±	21.56 ± 1.51^*^	<0.001	0.075	<0.001
			1.51^$^				
	Day 4	30.79 ± 1.15	21.88 ±	15.26 ± 1.15^*#^	<0.001	0.001	<0.001
			1.15^$^				
Escape distance (mm)	Day 1	150.37 ± 8.09	137.74 ±	117.36 ± 8.09^*^	0.023	0.236	0.625
			8.09				
	Day 2	141.34 ±6.83	119.55 ±	92.61 ±6.83^*#^	<0.001	0.028	0.094
			6.83				
	Day 3	115.56 ± 5.92	107.97 ±	67.9 ± 5.92^*#^	<0.001	<0.001	0.753
			5.92				
	Day 4	98.69 ±4.65	88.19 ±4.65	55.5 ±4.65^*#^	<0.001	<0.001	0.324
Crossing platform times	Day 5	5.63 ± 1.63	6.60 ± 2.60	8.31 ± 1.51^*^	0.004	0.243	0.692
Crossing target quadrant time (s)	Day 5	18.83 ±2.84	20.49 ±2.78	24.92 ±3.36^*#^	<0.001	0.003	0.227

Data are presented as the mean ± SD (*n* = 10/group). Statistical comparisons were performed using one-way analysis of variance followed with least significant difference post hoc test (crossing target quadrant time) or Welch test with Dunnett post hoc test (crossing platform times), and repeated measures analysis of variance followed with the Sidak post hoc test (escape latency and escape distance). The * represents a statistically significant difference between the NSC + silk and HIE groups, # represents a statistically significant difference between the NSC + silk and NSC groups, and $ represents a statistically significant difference between the NSC and HIE groups. HIE: Hypoxic-ischemic encephalopathy; NSC: neural stem cell.

These results suggest that hNSC treatment significantly improved motor function following HIE but had no effect on long-term cognitive function. The combination of hNSCs with ordered SF hydrogel did not offer an advantage in motor function improvement over hNSC treatment alone but did enhance cognitive recovery, leading to greater improvements in long-term spatial orientation and memory.

## Discussion

In this study, we investigated the ability of an ordered SF hydrogel to enhance hNSC-induced neural differentiation and synaptic development. We found that treatment with an hNSC–SF hydrogel complex resulted in cognitive improvement in rats subjected to HIE.

Untreated SF has limited medical value because of its lack of bioactive properties, while SF-derived electrospun materials have great potential for promoting tissue regeneration and in controlled drug delivery (Zou et al., 2021; Dos Santos et al., 2024). In the presence of an electric field, negatively charged silk nanofibers form a hydrogel at the positive electrode through electrostatic attraction, with the nanofibers intertwining to create a nano-oriented layered structure due to electrostatic repulsion (Liu et al., 2021). Compared with natural SF fibers, bundles of nanofibrils aligned along their long axis are highly bioactive and significantly enhance the growth, fiber spreading, and targeted migration of stem cells, while preserving both fiber morphology and connectivity (Fan et al., 2021; Zhu et al., 2024). Other directional continuous nanofiber platforms have been shown to promote neural differentiation of stem cells (Liu and Hu, 2018; Polo et al., 2021) and facilitate rapid, directional neurite growth (Silantyeva et al., 2018; Ghollasi and Poormoghadam, 2022). In this study, we prepared clinical-grade hNSCs as previously described (Lv et al., 2023) and confirmed their identity by the expression of NSC-specific markers, stable proliferation, and appropriate differentiation. Consistent with previous studies, we found that an ordered hydrogel material generated from a 1% SF solution promoted hNSC differentiation into neurons and inhibited hNSC differentiation into astrocytes.

SF has shown remarkable efficacy in spinal cord injury repair by enhancing axonal regeneration and promoting neural circuit formation (Sun et al., 2025). Seidlits et al. reported that 3D hyaluronic acid–based biomaterials promote the expansion and differentiation of neural stem/progenitor cells into neurons that express synaptophysin and exhibit electrophysiological characteristics typical of immature neurons, while inhibiting long-term astrocyte activation (Seidlits et al., 2019). In line with this, we also observed that an ordered SF hydrogel enhanced neuronal regeneration and network formation, as evidenced by increased axon length and synaptic protein expression in differentiated hNSCs.

Survivors of hypoxic-ischemic brain injury often experience motor dysfunction and cognitive decline, which are linked to disruptions in brain tissue architecture, interhemispheric connections, synaptic activity, and the overall neural circuitry (Murphy and Corbett, 2009; Newport et al., 2022; O’Sullivan et al., 2022; Wang et al., 2025). Cognitive and motor recovery following ischemic stroke can be achieved by promoting neuroplasticity (Xing and Bai, 2020). Stem cell therapy has shown promising results in the regulation of neural circuits (Luo et al., 2023; Yu et al., 2024). Transplantation of exogenous neuroepithelial stem cells or cortical organoids into the cortex of adult rats with stroke-induced injury improved neurological deficits and promoted both morphological and functional synaptic connections with host cortical neurons, facilitating incoming and outgoing transmission, as evidenced by immuno-electron microscopy and patch-clamp recordings (Yu et al., 2019; Grønning Hansen et al., 2020). In our study, NSCs promoted repair of HIE injury in a rat model, as evidenced by a reduction in infarct size, decreased astrocyte activation, and increased neuronal regeneration in the cerebral cortex and hippocampus, all of which contributed to improved motor and cognitive function.

Following CNS injury, the microenvironment deteriorates rapidly, progressively hindering nerve repair. This deterioration is characterized by reduced neurotrophic factor secretion, glial scar formation, and the presence of inhibitors of neuronal regeneration (Yang et al., 2020). Interestingly, ordered SF hydrogels create customizable microenvironments that optimize the differentiation and paracrine signaling of seeded cells, while also reducing inflammatory responses and immune rejection (Omidian and Chowdhury, 2023; Dai et al., 2024; Zhu et al., 2024). Carvalho et al. (2021) demonstrated that SF conduits loaded with NGF and glial cell line–derived neurotrophic factor provide enhanced neuroprotection and promote motor nerve reinnervation. Promsuk et al. (2024) showed that enhancing the mechanical stability of SF hydrogels contributes to the controlled release of growth factors and other protein components. Park et al. (2002) and Jin et al. (2010) reported that implanting an NSC–polymer scaffold complex into a cortical infarct cavity in rats reduced cavity size, promoted the survival of transplanted cells, resulted in the reconstitution of anatomical connections, with some cells differentiating into neurons and exhibiting electrophysiological properties. Davaa et al. (2023) found that NSCs implanted with an SF hydrogel were more likely to migrate into surrounding tissues, secrete neurotrophic factors, and enhance neural regeneration and lesion recovery than NSCs alone. Consistent with these findings, we observed elevated levels of BDNF and NGF expression when hNSCs were seeded on an ordered SF hydrogel. These increases were largely attributed to the hNSCs rather than the SF hydrogel because increasing the hydrogel concentration did not further elevate BDNF and NGF levels. Notably, while the hNSC–silk hydrogel complex did not enhance neuron regeneration or motor function, it significantly improved cognitive recovery, with greater improvements seen in spatial orientation and memory compared with hNSC treatment alone. Growth factors promote vascular remodeling and recovery of neurovascular function after ischemic stroke (Fang et al., 2023), ultimately aiding in cognitive recovery (Love and Miners, 2017; Hase et al., 2024). Zhou et al. (2023) reported that stem cells overexpressing BDNF effectively activate neuroprotective pathways, such as BDNF/TrkB signaling, modulate gene expression to promote neuroprotection and inhibit inflammation, and improve stroke recovery. Although synaptic network connections were not fully explored in this study, we observed a rapid and significant increase BDNF and NGF levels early after treatment, highlighting the advantage of combining silk hydrogels with hNSCs for more effective cognitive improvement.

Although the long-term survival, migration, differentiation, and functional outcomes of NSCs transplanted into the ischemic murine brain have been documented (Smith et al., 2012; Tsupykov et al., 2014; Tennstaedt et al., 2015), the survival rate and behavior of NSCs loaded onto SF hydrogels and implanted in brain tissue still need to be investigated because the potential for tumorigenesis, chronic immune responses, or other complications has not been fully explored. Future studies should focus on longitudinal analyses to assess the sustained impact on neuronal network formation, functional recovery, and any potential adverse effects. Second, functional analysis of synaptic formation *in vivo*, using techniques such as long-term potentiation or postsynaptic current recordings, is needed to better evaluate the functionality of synaptic networks following NSC–silk hydrogel complex treatment. Furthermore, additional investigation is needed to explore the direct regulatory effect of SF on NSC secretion of neurotrophic factors, as well as to optimize the properties of SF nanomaterials—such as surface modification, degradation rate, and mechanical properties—to enhance neurotrophic factor expression and release, thereby maximizing their therapeutic efficacy. Finally, a silk-only control group should be incorporated in future studies to evaluate the specific contribution of the SF nanomaterial to the observed outcomes. This control would allow for a clearer distinction between the effects of NSCs and the influence of the SF hydrogel alone.

Collectively, the findings from our study demonstrate that loading hNSCs into an ordered nanostructured hydrogel—electrostatically generated from an SF aqueous solution—enhanced their ability to promote HIE injury repair. The SF hydrogel provided an organized spatial structure that supported hNSC differentiation into neurons, while promoting axonal extension and synapse formation. Implantation of the NSC–hydrogel complex into injured brain tissue resulted in increased hippocampal neurotrophic factor expression and improved recovery of learning and spatial memory in rats. These findings underscore the potential of ordered SF hydrogels as a scaffold to enhance the therapeutic efficacy of hNSCs in treating HIE.

## Additional files:

***Additional Table 1:*** *Scoring criteria for animal balance beam test.*

***Additional Table 2:*** *Balance beam scores in HIE rat models.*

***Additional Table 3:*** *Cylinder test statistics in HIE rat models. *

***Additional Table 4:*** *Morris water maze test results in HIE rat models.*

## Data Availability

*All data generated or analyzed during this study are included in this published article and its Additional files*.

## References

[R1] Bai S, Zhang X, Lu Q, Sheng W, Liu L, Dong B, Kaplan DL, Zhu H (2014). Reversible hydrogel-solution system of silk with high beta-sheet content. Biomacromolecules.

[R2] Boboc IKS, Rotaru-Zavaleanu AD, Calina D, Albu CV, Catalin B, Turcu-Stiolica A (2023). A preclinical systematic review and meta-analysis of behavior testing in mice models of ischemic stroke. Life (Basel).

[R3] Brooks SP, Dunnett SB (2009). Tests to assess motor phenotype in mice: a user’s guide. Nat Rev Neurosci.

[R4] Cadena M, Ning L, King A, Hwang B, Jin L, Serpooshan V, Sloan SA (2021). 3D bioprinting of neural tissues. Adv Healthc Mater.

[R5] Carvalho CR, Chang W, Silva-Correia J, Reis RL, Oliveira JM, Kohn J (2021). Engineering silk fibroin-based nerve conduit with neurotrophic factors for proximal protection after peripheral nerve injury. Adv Healthc Mater.

[R6] Daadi MM, Li Z, Arac A, Grueter BA, Sofilos M, Malenka RC, Wu JC, Steinberg GK (2009). Molecular and magnetic resonance imaging of human embryonic stem cell-derived neural stem cell grafts in ischemic rat brain. Mol Ther.

[R7] Daadi MM, Davis AS, Arac A, Li Z, Maag AL, Bhatnagar R, Jiang K, Sun G, Wu JC, Steinberg GK (2010). Human neural stem cell grafts modify microglial response and enhance axonal sprouting in neonatal hypoxic-ischemic brain injury. Stroke.

[R8] Dai S, Liang H, Zhu M, Zhang Y (2024). Electrospun silk for biomedical applications. Med-X.

[R9] Davaa G, Hong JY, Lee JH, Kim MS, Buitrago JO, Li YM, Lee HH, Han DW, Leong KW, Hyun JK, Kim HW (2023). Delivery of induced neural stem cells through mechano-tuned silk-collagen hydrogels for the recovery of contused spinal cord in rats. Adv Healthc Mater.

[R10] De Vitis E, Stanzione A, Romano A, Quattrini A, Gigli G, Moroni L, Gervaso F, Polini A (2024). The evolution of technology-driven in vitro models for neurodegenerative diseases. Adv Sci (Weinh).

[R11] Dos Santos FV, Siqueira RL, de Morais Ramos L, Yoshioka SA, Branciforti MC, Correa DS (2024). Silk fibroin-derived electrospun materials for biomedical applications: A review. Int J Biol Macromol.

[R12] Ebrahimi T, Abasi M, Seifar F, Eyvazi S, Hejazi MS, Tarhriz V, Montazersaheb S (2021). Transplantation of stem cells as a potential therapeutic strategy in neurodegenerative disorders. Curr Stem Cell Res Ther.

[R13] Ehlting A, Zweyer M, Maes E, Schleehuber Y, Doshi H, Sabir H, Bernis ME (2022). Impact of hypoxia-ischemia on neurogenesis and structural and functional outcomes in a mild-moderate neonatal hypoxia-ischemia brain injury model. Life (Basel).

[R14] Fan L, Li JL, Cai Z, Wang X (2021). Bioactive hierarchical silk fibers created by bioinspired self-assembly. Nat Commun.

[R15] Fang J, Wang Z, Miao CY (2023). Angiogenesis after ischemic stroke. Acta Pharmacol Sin.

[R16] Ghollasi M, Poormoghadam D (2022). Enhanced neural differentiation of human-induced pluripotent stem cells on aligned laminin-functionalized polyethersulfone nanofibers; a comparison between aligned and random fibers on neurogenesis. J Biomed Mater Res A.

[R17] Grønning Hansen M, Laterza C, Palma-Tortosa S, Kvist G, Monni E, Tsupykov O, Tornero D, Uoshima N, Soriano J, Bengzon J, Martino G, Skibo G, Lindvall O, Kokaia Z (2020). Grafted human pluripotent stem cell-derived cortical neurons integrate into adult human cortical neural circuitry. Stem Cells Transl Med.

[R18] Guo Q, Zhang J, Zheng Z, Li X, Wang F, Liu S (2020). Lentivirus-mediated microRNA-26a-modified neural stem cells improve brain injury in rats with cerebral palsy. J Cell Physiol.

[R19] Hase Y, Jobson D, Cheong J, Gotama K, Maffei L, Hase M, Hamdan A, Ding R, Polivkoski T, Horsburgh K, Kalaria RN (2024). Hippocampal capillary pericytes in post-stroke and vascular dementias and Alzheimer’s disease and experimental chronic cerebral hypoperfusion. Acta Neuropathol Commun.

[R20] He T, Yang GY, Zhang Z (2022). Crosstalk of astrocytes and other cells during ischemic stroke. Life (Basel).

[R21] Hol EM, Pekny M (2015). Glial fibrillary acidic protein (GFAP) and the astrocyte intermediate filament system in diseases of the central nervous system. Curr Opin Cell Biol.

[R22] Huang L, Zhang L (2019). Neural stem cell therapies and hypoxic-ischemic brain injury. Prog Neurobiol.

[R23] Jiang JP, Liu XY, Zhao F, Zhu X, Li XY, Niu XG, Yao ZT, Dai C, Xu HY, Ma K, Chen XY, Zhang S (2020). Three-dimensional bioprinting collagen/silk fibroin scaffold combined with neural stem cells promotes nerve regeneration after spinal cord injury. Neural Regen Res.

[R24] Jiang XC, Xiang JJ, Wu HH, Zhang TY, Zhang DP, Xu QH, Huang XL, Kong XL, Sun JH, Hu YL, Li K, Tabata Y, Shen YQ, Gao JQ (2019). Neural stem cells transfected with reactive oxygen species-responsive polyplexes for effective treatment of ischemic stroke. Adv Mater.

[R25] Jin K, Mao X, Xie L, Galvan V, Lai B, Wang Y, Gorostiza O, Wang X, Greenberg DA (2010). Transplantation of human neural precursor cells in Matrigel scaffolding improves outcome from focal cerebral ischemia after delayed postischemic treatment in rats. J Cereb Blood Flow Metab.

[R26] Kalladka D, Sinden J, Pollock K, Haig C, McLean J, Smith W, McConnachie A, Santosh C, Bath PM, Dunn L, Muir KW (2016). Human neural stem cells in patients with chronic ischaemic stroke (PISCES): a phase 1, first-in-man study. Lancet.

[R27] Kim JT, Cho SM, Youn DH, Hong EP, Park CH, Lee Y, Jung H, Jeon JP (2023). Therapeutic effect of a hydrogel-based neural stem cell delivery sheet for mild traumatic brain injury. Acta Biomater.

[R28] Koffler J, Zhu W, Qu X, Platoshyn O, Dulin JN, Brock J, Graham L, Lu P, Sakamoto J, Marsala M, Chen S, Tuszynski MH (2019). Biomimetic 3D-printed scaffolds for spinal cord injury repair. Nat Med.

[R29] Lee IS, Koo KY, Jung K, Kim M, Kim IS, Hwang K, Yun S, Lee H, Shin JE, Park KI (2017). Neurogenin-2-transduced human neural progenitor cells attenuate neonatal hypoxic-ischemic brain injury. Transl Res.

[R30] Li C, Kuss M, Kong Y, Nie F, Liu X, Liu B, Dunaevsky A, Fayad P, Duan B, Li X (2021). 3D printed hydrogels with aligned microchannels to guide neural stem cell migration. ACS Biomater Sci Eng.

[R31] Liaudanskaya V, Jgamadze D, Berk AN, Bischoff DJ, Gu BJ, Hawks-Mayer H, Whalen MJ, Chen HI, Kaplan DL (2019). Engineering advanced neural tissue constructs to mitigate acute cerebral inflammation after brain transplantation in rats. Biomaterials.

[R32] Liu H, Ming J, Guo X, Huang X, Zuo B, Ning X (2021). Low voltage electric field governs fibrous silk electrogels. Mater Des.

[R33] Liu Z, Hu Z (2018). Aligned contiguous microfiber platform enhances neural differentiation of embryonic stem cells. Sci Rep.

[R34] Liu Z, Wang Z, Zhu Z, Hong J, Cui L, Hao Y, Cheng G, Tan R (2023). Crocetin regulates functions of neural stem cells to generate new neurons for cerebral ischemia recovery. Adv Healthc Mater.

[R35] Livak KJ, Schmittgen TD (2001). Analysis of relative gene expression data using real-time quantitative PCR and the 2(-Delta Delta C(T)) Method. Methods.

[R36] Love S, Miners JS (2017). Small vessel disease, neurovascular regulation and cognitive impairment: post-mortem studies reveal a complex relationship, still poorly understood. Clin Sci (Lond).

[R37] Luo BY, Zhou HS, Sun YF, Xiao QX, Chen L, She HQ, Wang SF, Yan SS, Chang QY, He YQ, Xiong LL (2023). The fate and prospects of stem cell therapy in the treatment of hypoxic-ischemic encephalopathy. Eur J Neurosci.

[R38] Lv Z, Li Y, Wang Y, Cong F, Li X, Cui W, Han C, Wei Y, Hong X, Liu Y, Ma L, Jiao Y, Zhang C, Li H, Jin M, Wang L, Ni S, Liu J (2023). Safety and efficacy outcomes after intranasal administration of neural stem cells in cerebral palsy: a randomized phase 1/2 controlled trial. Stem Cell Res Ther.

[R39] Mattiassi S, Conner AA, Feng F, Goh ELK, Yim EKF (2023). The combined effects of topography and stiffness on neuronal differentiation and maturation using a hydrogel platform. Cells.

[R40] Muir KW, Bulters D, Willmot M, Sprigg N, Dixit A, Ward N, Tyrrell P, Majid A, Dunn L, Bath P, Howell J, Stroemer P, Pollock K, Sinden J (2020). Intracerebral implantation of human neural stem cells and motor recovery after stroke: multicentre prospective single-arm study (PISCES-2). J Neurol Neurosurg Psychiatry.

[R41] Murphy TH, Corbett D (2009). Plasticity during stroke recovery: from synapse to behaviour. Nat Rev Neurosci.

[R42] Newport EL, Seydell-Greenwald A, Landau B, Turkeltaub PE, Chambers CE, Martin KC, Rennert R, Giannetti M, Dromerick AW, Ichord RN, Carpenter JL, Berl MM, Gaillard WD (2022). Language and developmental plasticity after perinatal stroke. Proc Natl Acad Sci U S A.

[R43] O’Sullivan MJ, Oestreich LKL, Wright P, Clarkson AN (2022). Cholinergic and hippocampal systems facilitate cross-domain cognitive recovery after stroke. Brain.

[R44] Omidian H, Chowdhury SD (2023). Advancements and applications of injectable hydrogel composites in biomedical research and therapy. Gels.

[R45] Park KI, Teng YD, Snyder EY (2002). The injured brain interacts reciprocally with neural stem cells supported by scaffolds to reconstitute lost tissue. Nat Biotechnol.

[R46] Polo Y, Luzuriaga J, Iturri J, Irastorza I, Toca-Herrera JL, Ibarretxe G, Unda F, Sarasua JR, Pineda JR, Larrañaga A (2021). Nanostructured scaffolds based on bioresorbable polymers and graphene oxide induce the aligned migration and accelerate the neuronal differentiation of neural stem cells. Nanomedicine.

[R47] Promsuk J, Manissorn J, Laomeephol C, Luckanagul JA, Methachittipan A, Tonsomboon K, Jenjob R, Yang SG, Thongnuek P, Wangkanont K (2024). Optimizing protein delivery rate from silk fibroin hydrogel using silk fibroin-mimetic peptides conjugation. Sci Rep.

[R48] Salikhova D, Bukharova T, Cherkashova E, Namestnikova D, Leonov G, Nikitina M, Gubskiy I, Akopyan G, Elchaninov A, Midiber K, Bulatenco N, Mokrousova V, Makarov A, Yarygin K, Chekhonin V, Mikhaleva L, Fatkhudinov T, Goldshtein D (2021). Therapeutic effects of hiPSC-derived glial and neuronal progenitor cells-conditioned medium in experimental ischemic stroke in rats. Int J Mol Sci.

[R49] Santos AK, Gomes KN, Parreira RC, Scalzo S, Pinto MCX, Santiago HC, Birbrair A, Sack U, Ulrich H, Resende RR (2022). Mouse neural stem cell differentiation and human adipose mesenchymal stem cell transdifferentiation into neuron- and oligodendrocyte-like cells with myelination potential. Stem Cell Rev Rep.

[R50] Seidlits SK, Liang J, Bierman RD, Sohrabi A, Karam J, Holley SM, Cepeda C, Walthers CM (2019). Peptide-modified, hyaluronic acid-based hydrogels as a 3D culture platform for neural stem/progenitor cell engineering. J Biomed Mater Res A.

[R51] Semmler L, Naghilou A, Millesi F, Wolf S, Mann A, Stadlmayr S, Mero S, Ploszczanski L, Greutter L, Woehrer A, Placheta-Györi E, Vollrath F, Weiss T, Radtke C (2023). Silk-in-silk nerve guidance conduits enhance regeneration in a rat sciatic nerve injury model. Adv Healthc Mater.

[R52] Silantyeva EA, Nasir W, Carpenter J, Manahan O, Becker ML, Willits RK (2018). Accelerated neural differentiation of mouse embryonic stem cells on aligned GYIGSR-functionalized nanofibers. Acta Biomater.

[R53] Singh A, Shiekh PA, Das M, Seppälä J, Kumar A (2019). Aligned chitosan-gelatin cryogel-filled polyurethane nerve guidance channel for neural tissue engineering: Fabrication, characterization, and in vitro evaluation. Biomacromolecules.

[R54] Smith EJ, Stroemer RP, Gorenkova N, Nakajima M, Crum WR, Tang E, Stevanato L, Sinden JD, Modo M (2012). Implantation site and lesion topology determine efficacy of a human neural stem cell line in a rat model of chronic stroke. Stem Cells.

[R55] Sun J, Ru M, Du M, Wang L, Yan S, Zhang Q (2025). Silk-based biomaterials for promoting spinal cord regeneration: A review. Int J Biol Macromol.

[R56] Sun X, Zhang C, Xu J, Zhai H, Liu S, Xu Y, Hu Y, Long H, Bai Y, Quan D (2020). Neurotrophin-3-loaded multichannel nanofibrous scaffolds promoted anti-inflammation, neuronal differentiation, and functional recovery after spinal cord injury. ACS Biomater Sci Eng.

[R57] Tan KK, Tann JY, Sathe SR, Goh SH, Ma D, Goh EL, Yim EK (2015). Enhanced differentiation of neural progenitor cells into neurons of the mesencephalic dopaminergic subtype on topographical patterns. Biomaterials.

[R58] Tang X, Deng P, Li L, He Y, Wang J, Hao D, Yang H (2024). Advances in genetically modified neural stem cell therapy for central nervous system injury and neurological diseases. Stem Cell Res Ther.

[R59] Tennstaedt A, Aswendt M, Adamczak J, Collienne U, Selt M, Schneider G, Henn N, Schaefer C, Lagouge M, Wiedermann D, Kloppenburg P, Hoehn M (2015). Human neural stem cell intracerebral grafts show spontaneous early neuronal differentiation after several weeks. Biomaterials.

[R60] Tsupykov O, Kyryk V, Smozhanik E, Rybachuk O, Butenko G, Pivneva T, Skibo G (2014). Long-term fate of grafted hippocampal neural progenitor cells following ischemic injury. J Neurosci Res.

[R61] Vannucci RC, Vannucci SJ (2005). Perinatal hypoxic-ischemic brain damage: Evolution of an animal model. Dev Neurosci.

[R62] Waites CL, Leal-Ortiz SA, Okerlund N, Dalke H, Fejtova A, Altrock WD, Gundelfinger ED, Garner CC (2013). Bassoon and Piccolo maintain synapse integrity by regulating protein ubiquitination and degradation. EMBO J.

[R63] Wang Y, Li Y, Gu Y, Ma W, Guan Y, Guo M, Shao Q, Ji X, Liu J (2025). Decreased levels of phosphorylated synuclein in plasma are correlated with poststroke cognitive impairment. Neural Regen Res.

[R64] Wei G, Wang J, Lv Q, Liu M, Xu H, Zhang H, Jin L, Yu J, Wang X (2018). Three-dimensional coculture of primary hepatocytes and stellate cells in silk scaffold improves hepatic morphology and functionality in vitro. J Biomed Mater Res A.

[R65] Wu H, Fang Q, Liu J, Yu X, Xu Y, Wan Y, Xiao B (2018). Multi-tubule conduit-filler constructs loaded with gradient-distributed growth factors for neural tissue engineering applications. J Mech Behav Biomed Mater.

[R66] Xing Y, Bai Y (2020). A review of exercise-induced neuroplasticity in ischemic stroke: pathology and mechanisms. Mol Neurobiol.

[R67] Xu C, Wu P, Yang K, Mu C, Li B, Li X, Wang Z, Liu Z, Wang X, Luo Z (2024). Multifunctional biodegradable conductive hydrogel regulating microenvironment for stem cell therapy enhances the nerve tissue repair. Small.

[R68] Xu Y, Zhou J, Liu C, Zhang S, Gao F, Guo W, Sun X, Zhang C, Li H, Rao Z, Qiu S, Zhu Q, Liu X, Guo X, Shao Z, Bai Y, Zhang X, Quan D (2021). Understanding the role of tissue-specific decellularized spinal cord matrix hydrogel for neural stem/progenitor cell microenvironment reconstruction and spinal cord injury. Biomaterials.

[R69] Yang L, Conley BM, Cerqueira SR, Pongkulapa T, Wang S, Lee JK, Lee KB (2020). Effective modulation of CNS inhibitory microenvironment using bioinspired hybrid-nanoscaffold-based therapeutic interventions. Adv Mater.

[R70] Yang M, Wang K, Liu B, Shen Y, Liu G (2025). Hypoxic-ischemic encephalopathy: pathogenesis and promising therapies. Mol Neurobiol.

[R71] Yonesi M, Garcia-Nieto M, Guinea GV, Panetsos F, Pérez-Rigueiro J, González-Nieto D (2021). Silk fibroin: An ancient material for repairing the injured nervous system. Pharmaceutics.

[R72] Yu J, Chen G, Zhu H, Zhong Y, Yang Z, Jian Z, Xiong X (2024). Metabolic and proteostatic differences in quiescent and active neural stem cells. Neural Regen Res.

[R73] Yu SP, Tung JK, Wei ZZ, Chen D, Berglund K, Zhong W, Zhang JY, Gu X, Song M, Gross RE, Lin SZ, Wei L (2019). Optochemogenetic stimulation of transplanted iPS-NPCs enhances neuronal repair and functional recovery after ischemic stroke. J Neurosci.

[R74] Zhou X, Deng X, Liu M, He M, Long W, Xu Z, Zhang K, Liu T, So KF, Fu QL, Zhou L (2023). Intranasal delivery of BDNF-loaded small extracellular vesicles for cerebral ischemia therapy. J Control Release.

[R75] Zhu JC, Wang H, Wu CX, Zhang KQ, Ye H (2024). Tailoring silk fibroin fibrous architecture by a high-yield electrospinning method for fast wound healing possibilities. Biotechnol Bioeng.

[R76] Zou S, Wang X, Fan S, Yao X, Zhang Y, Shao H (2021). Electrospun regenerated Antheraea pernyi silk fibroin scaffolds with improved pore size, mechanical properties and cytocompatibility using mesh collectors. J Mater Chem B.

